# Supplementary figures and images for: Recombinant 60-kDa heat shock protein from Paracoccidioides brasiliensis induces the death of mouse lymphocytes in a mechanism dependent on Toll-like receptor 4 and tumor necrosis factor
Source: PLoS One. 2024 Mar 21;19(3):e0300364. doi: 10.1371/journal.pone.0300364 (PMC10956883; doi:10.1371/journal.pone.0300364)

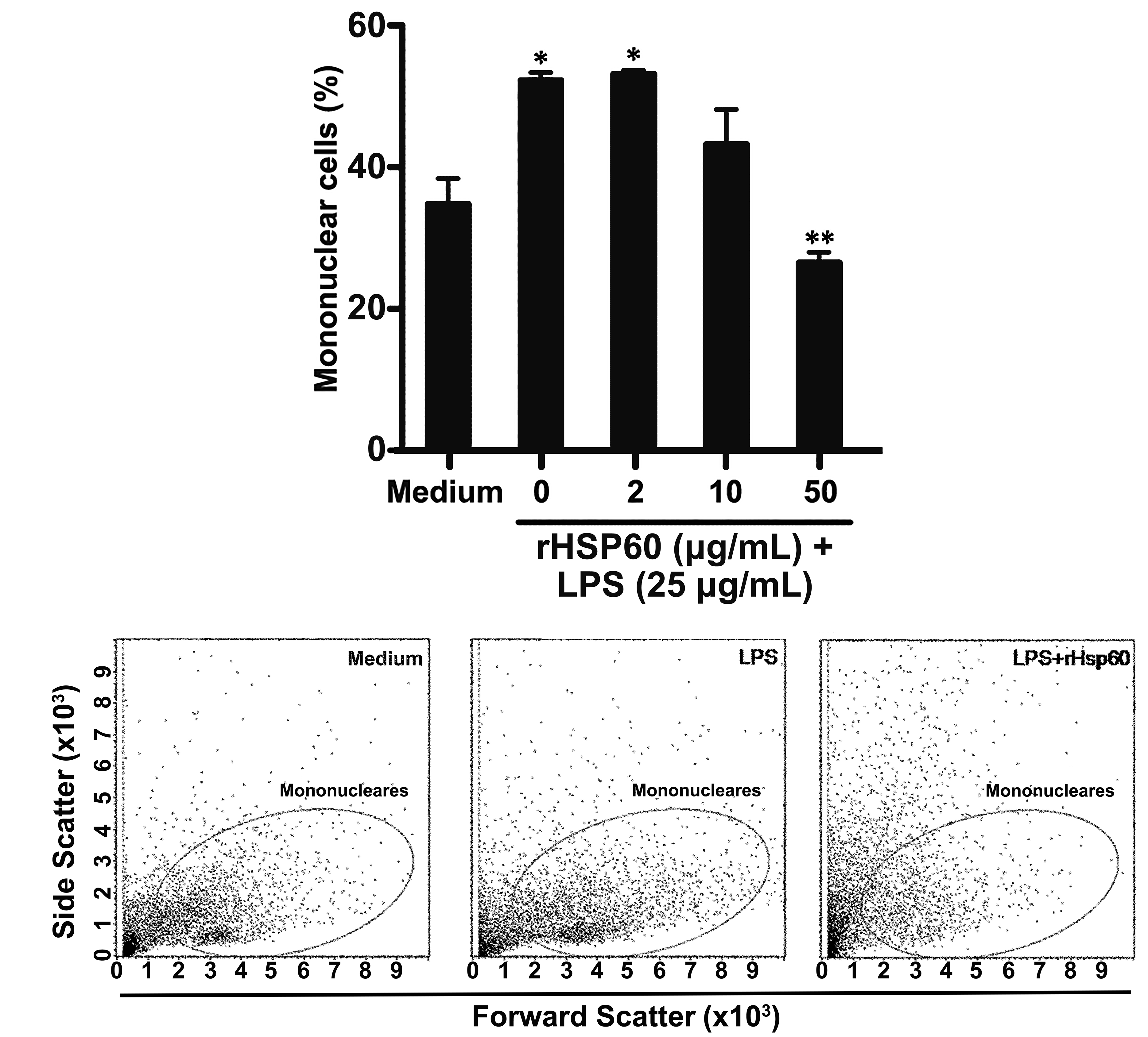

Supplement: S1 Fig — Spleen cells (4.5 × 10^5^ cells/mL) from WT mice were stained with CFSE and stimulated with only LPS at 25 μg/mL (0) or LPS plus 2, 10, or 50 μg/mL of rHSP60 for 24 hours. The negative control consisted of unstimulated cells (medium). The cells were acquired in a Guava Cytometer and analyzed in FlowJo software. Bars represent means ± SD of cell percentage. *P < 0.05 compared with unstimulated cells (Medium) and cells stimulated with rHSP60 at 50 μg/mL. **P < 0.05 compared with other groups. Experiment representative of two experiments. (TIF) [file pone.0300364.s001.tif]

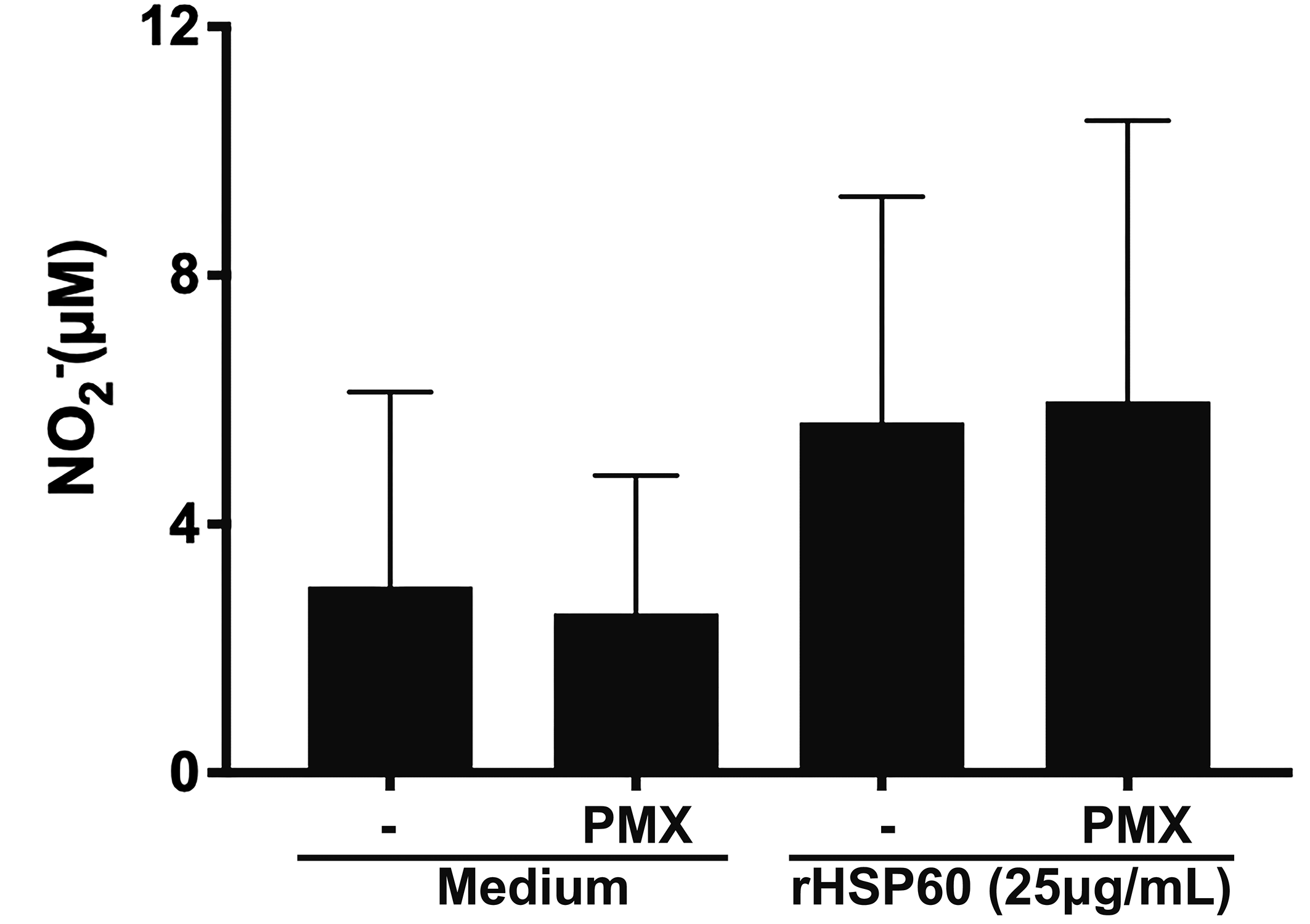

Supplement: S2 Fig — Spleen cells (4.5 × 10^5^ cells/mL) from WT mice were stimulated with 25 μg/mL of rHSP60 for 24 hours in the presence or absence of 30 μg/mL of polymyxin. The negative control consisted of unstimulated cells (medium). The supernatants were collected and the NO2- determined with Griess reagent. Bars represent means ± SD of NO2- concentrations. Experiment representative of two experiments. (TIF) [file pone.0300364.s002.tif]

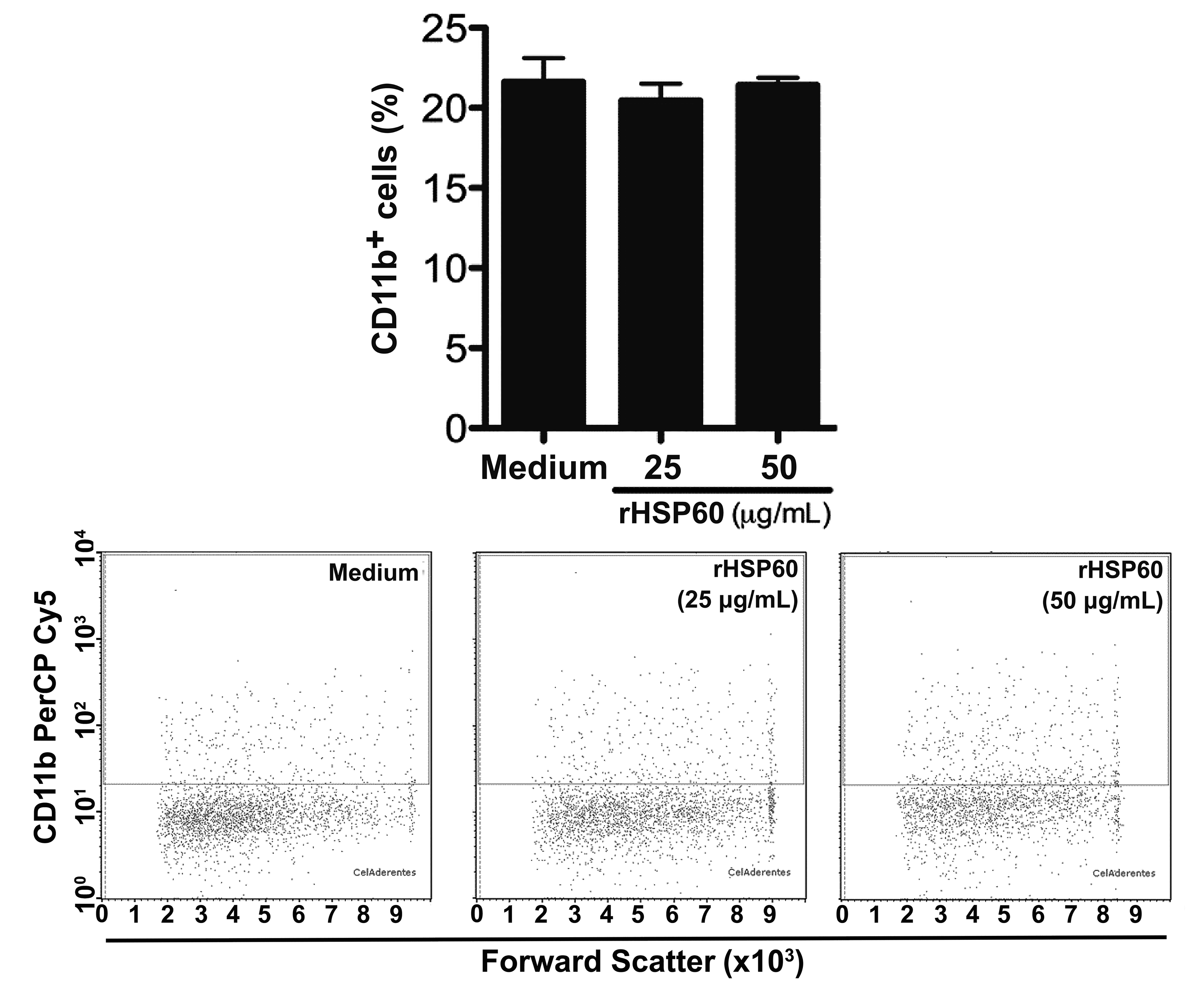

Supplement: S3 Fig — Adherent spleen cells (4.5 × 10^5^ cells/mL) from WT mice were stimulated with rHSP60 at 25 or 50 μg/mL for 24 hours. The negative control consisted of unstimulated cells (Medium). The cells were labeled with PerCP-Cy5.5-conjugated anti-CD11b antibodies, acquired in a Guava Cytometer, and analyzed in FlowJo software. Bars represent means ± SD of cell percentage. Experiment representative of two experiments. (TIF) [file pone.0300364.s003.tif]
