# Supplementary material for: Recombinant 60-kDa heat shock protein from Paracoccidioides brasiliensis induces the death of mouse lymphocytes in a mechanism dependent on Toll-like receptor 4 and tumor necrosis factor
Source: PLoS One. 2024 Mar 21;19(3):e0300364. doi: 10.1371/journal.pone.0300364 (PMC10956883; doi:10.1371/journal.pone.0300364)
Supplement: Attachment — Submitted filename: Response to reviewers.docx [file pone.0300364.s004.docx]

**Point-by-point Responses to the ReviewerS’ COMMENTS AND JOURNAL REQUIREMENTS**

**Journal requirements**

1. Please ensure that your manuscript meets PLoS ONE's style requirements, including those for file naming.

***Answer:*** *The manuscript was entirely reviewed.*

2. To comply with PLOS ONE submissions requirements, in your Methods section, please provide additional information regarding the experiments involving animals and ensure you have included details on (1) methods of sacrifice, (2) methods of anesthesia and/or analgesia, and (3) efforts to alleviate suffering.

***Answer:*** *We added the following information on page 4, lines 88-94,*

*Mice were acclimated to* the facility for one week before initiating the experiment, housed in individually ventilated *microisolators*, light-tight cabinets (Alesco, Capivari, Brazil), maintained at 20–22°C, a 12 h light-dark cycle, and with access to chow and water ad libitum. We cleaned all microisolators twice a week and bedded them with autoclaved softwood shavings. *Mice were maintained under specific pathogen-free conditions. For all immunization and euthanasia approaches, mice were anesthetized by intraperitoneal injection of ketamine (100 mg/kg) and xylazine (8 mg/kg) mixture.*

*page 6, lines 122 and 123,*

*The animals were anesthetized as described above and submitted to euthanasia by cervical dislocation 7 days after immunization.*

*page 6, lines 141and 142, and*

*… animals were submitted to euthanasia as described above and had the spleen* aseptically removed and dissociated. The cell *…*

*page 8, lines 172-174.*

*WT mice were injected intramuscularly (i.m.) once with 50 μg/mL of rHSP60 or thrice in 15-day intervals between doses. Seven days after the only or last dose of rHSP60, the animals were euthanized as described above and had the spleen removed.*

3. Thank you for stating the following financial disclosure:

“The São Paulo Research Foundation [grant numbers 2017/01390-8, and 2021/01528-5], and CAPES.”

Please state what role the funders took in the study. If the funders had no role, please state: "The funders had no role in study design, data collection and analysis, decision to publish, or preparation of the manuscript."

If this statement is not correct you must amend it as needed.

Please include this amended Role of Funder statement in your cover letter; we will change the online submission form on your behalf.

***Answer:*** *We included the Role of Funder* statement *in the cover letter.*

4. Thank you for stating the following in the Acknowledgments Section of your manuscript:

“The authors thank Mrs. Denise B. Ferraz and Dr. Taise N. Landgraf for their excellent technical assistance in cytometry, and Dr. João Santana da Silva from the Oswaldo Cruz Foundation, Ribeirão Preto, who provided us with A20 and J774 cell lines. This work was supported by the São Paulo Research Foundation – FAPESP [grant numbers 2017/01390-8, and 2021/01528-5].”

We note that you have provided funding information that is currently declared in your Funding Statement. However, funding information should not appear in the Acknowledgments section or other areas of your manuscript. We will only publish funding information present in the Funding Statement section of the online submission form.

Please remove any funding-related text from the manuscript and let us know how you would like to update your Funding Statement. Currently, your Funding Statement reads as follows:

“The São Paulo Research Foundation [grant numbers 2017/01390-8, and 2021/01528-5], and CAPES.”

Please include your amended statements within your cover letter; we will change the online submission form on your behalf.

***Answer:*** *We removed the funding-related text from the manuscript's Acknowledgments section.*

5. We note that you have included the phrase “data not shown” in your manuscript. Unfortunately, this does not meet our data sharing requirements. PLoS does not permit references to inaccessible data. We require that authors provide all relevant data within the paper, Supporting Information files, or in an acceptable, public repository. Please add a citation to support this phrase or upload the data that corresponds with these findings to a stable repository (such as Figshare or Dryad) and provide and URLs, DOIs, or accession numbers that may be used to access these data. Or, if the data are not a core part of the research being presented in your study, we ask that you remove the phrase that refers to these data.

***Answer*:** *Thank you. As asked, we changed the manuscript.*

*On page 9, lines 200-202, the data not shown were very similar to the one shown, so we modified the following.*

*“Even when we tested these cells with rHSP60 at a concentration of 25 or 50 μg/mL, the results were similar to stimulation with 2 and 10 μg/mL of rHSP60.”*

On page 9, line 206, we think that Fig 1B could replace the data not shown.

On page 9, lines 216, and page 13, line 306, we added a supporting figure (S1 Fig).

On pages 11, lines 261, and page 18, line 425, we added a supporting figure (S3 Fig).

On page 13, line 311, “data not shown” was replaced by a reference.

On page 17, line 414, we added a supporting figure (S2 Fig), which was also added to a new sentence in the results (on page 10, lines 228-229).

6. Please review your reference list to ensure that it is complete and correct. If you have cited papers that have been retracted, please include the rationale for doing so in the manuscript text, or remove these references and replace them with relevant current references. Any changes to the reference list should be mentioned in the rebuttal letter that accompanies your revised manuscript. If you need to cite a retracted article, indicate the article’s retracted status in the References list and also include a citation and full reference for the retraction notice.

***Answer:*** *The references were reviewed.*

**Response to reviewers**

**Reviewer #1:**

My major concern regards the great number of "Data not shown" in the manuscript. These data should be provided as part of the manuscript or its supporting information.

***Answer*:** *Thank you. As asked, we changed the manuscript, providing some data and modifying the text to become clearer.*

*On page 9, lines 200-202, the data not shown were very similar to the one shown, so we modified the following.*

*“Even when we tested these cells with rHSP60 at a concentration of 25 or 50 μg/mL, the results were similar to stimulation with 2 and 10 μg/mL of rHSP60.”*

On page 9, line 206, we think that Fig 1B could replace the data not shown.

On page 9, lines 216, and page 13, line 306, we added a supporting figure (S1 Fig).

On pages 11, lines 261, and page 18, line 425, we added a supporting figure (S3 Fig).

On page 13, line 311, “data not shown” was replaced by a reference.

On page 17, line 414, we added a supporting figure (S2 Fig), which was also added to a new sentence in the results (on page 10, lines 228-229).

**Reviewer #2:**

Page 3, line 49: Please correct the sentence “…the lungs.t cases registered, about 85%, held in Brazil…”

***Answer:*** *Thank you. The mistake has been fixed.*

*… the lungs, and about 85% of registered cases have occurred in Brazil [1-3].*

Page 7, lines 161 and 162: Please correct the sentence “…last dose spleen of animals was euthanized and had the spleen removed.”

***Answer:*** *Thank you. The mistake has been fixed. Now, on page 8, lines 173 and 174.*

“*Seven days after the only or last dose of rHSP60, the animals were euthanized as described above and had the spleen removed.*”

Page 8, line 185: Please correct the formation of the word “and” in the title “rHSP60 inhibits the viability and proliferation of lymphocytes”

***Answer:*** *Thank you. The mistake has been fixed. Now, on page 9, line 197.*

Legend of figure 2, page 11, line 252: Please put in bold the letter “B” in the sentence “B Results are presented as mean…”

***Answer:*** *Thank you. The mistake has been fixed. Now, on page 11, line 266.*

Page 12, line 271: Please explain whether the group control is the same of cells treated with medium only. If they are the same please uniformize the name of the groups in the legends and graphics.

***Answer:*** Thank you. We reviewed all the text. Now, on page 12, line 285.

Legend of figure 5, page 14: Please inform the number of replications in this experiment. ***Answer:*** Thank you. The number of replications was shown. Now, on page 15, line 352.

Figures 1A, 2A, and C, 6A: Please increase the quality of them. The words are blurred.

***Answer:*** Thank you. We improved the figure quality.

Figure 1B: Please correct the numbers at the Y-axis, the number 100 appears twice.

***Answer:*** Thank you. The mistake was corrected.
